# HIV infected men who have sex with men in Israel: knowledge, attitudes and sexual behavior

**DOI:** 10.1186/s12879-017-2782-1

**Published:** 2017-10-12

**Authors:** Zohar Mor, Dan Turner, Yuval Livnat, Itzchak Levy

**Affiliations:** 1 Tel Aviv Department of Health, 12 Ha’arba’a St, 6473912 Tel Aviv, Israel; 2 Faculty of Medicine, Tel Aviv University, Tel Aviv, Israel; 3 AIDS Treatment Center, Sourasky Medical Center, Tel Aviv, Israel; 4 Israeli AIDS Task Force, Tel Aviv, Israel; 5 AIDS Treatment Center, Sheba Medical Center, Ramat Gan, Israel

**Keywords:** Aids, Disclosure, Gay men, Sero-discordant, Unprotected anal intercourse

## Abstract

**Background:**

HIV-infected (HIVI) men who have sex with men (MSM) may transmit HIV to their sero-discordant sex partner/s. This study assesses the knowledge, attitudes and sex-practices of Israeli HIVI-MSM.

**Methods:**

This cross-sectional study compared HIVI-MSM to self-reported HIV-uninfected (HIVU) MSM by using anonymous questionnaires that were distributed in AIDS-treatment centers and gay-related internet-sites in 2015. Unprotected anal intercourse (UAI) in the last 6 months was the outcome variable.

**Results:**

Of 300 HIVI-MSM and 1299 HIVU-MSM, UAI with sero-discordant/unknown-status partner/s was performed by 12.1% and 17.9%, respectively, *p*=0.02. UAI with sero-discordant/unknown-status among HIVI-MSM and HIVU-MSM was associated with the type of partnership: 37.7% *vs*. 52.4% for steady partner/s, 19.0% *vs*. 39.9% for sex-buddies and 23.5% *vs*. 24.0% for casual partner/s (*p*<0.001, *p*=0.01, *p*=0.6), respectively. On these occasions, HIVI-MSM were more likely to be receptive during UAI: 92.3%, 87.5% and 83.3% for steady partner/s, sex buddies and casual partner/s, respectively. In cases HIVI-MSM performed UAI, 31.3% expected their partner/s to share responsibility for condom-use *vs*. 9.7% of HIVU-MSM.

HIVI-MSM were involved in risky sexual-behaviors, such as substances-use, earlier sexual debut and sex for money. HIVI-MSM were more likely to disclose their HIV-status with their partner before sex and demonstrated better knowledge about HIV-transmission than HIVU-MSM.

**Conclusion:**

HIVI-MSM performed UAI with sero-discordant/unknown-status partner/s less frequently than HIVU-MSM. Their condom-use practices were associated with the type of partner, and were lower for casual *vs*. steady partners or sex-buddies. HIVI-MSM tended to use sero-adaptive strategies to reduce the potential risk of HIV-transmission to their sero-discordant/unknown-status partner/s.

**Electronic supplementary material:**

The online version of this article (10.1186/s12879-017-2782-1) contains supplementary material, which is available to authorized users.

## Background

The burden of HIV/AIDS in Israel has increased during the last 15 years, especially among men who have sex with men (MSM), who comprise ~40% of all males infected with HIV [1], and it is estimated that the prevalence of HIV among MSM in 2011 was 0.7–1.0% [2]. This trend is related to the declining death rate among HIV-infected individuals due to the beneficial outcomes of the anti-retroviral treatment (ART). The predicted life expectancy has improved dramatically and is now only ~8 years shorter than that of non-HIV infected individuals [3]. The success of ART has also changed the perception of HIV/AIDS-infection from a lethal disease to a chronic condition.

The beneficial effects of ART provide HIV-infected MSM a full and relatively healthy life, while keeping their social circles preserved. They therefore have opportunities to maintain a sex network, while being able to potentially transmit the infection to other healthy sex partners. Some HIV-infected MSM who achieve undetectable viral load level may decide to perform unprotected anal sex (UAI), considering themselves as not being able to transmit the virus [4]. Other HIV-infected MSM may perform UAI with their sex partner/s following a disclosure that they are HIV-infected - a phenomenon called ‘sero-sorting’ [5]. In parallel, the perceived risk of HIV infection in the gay community has declined, which may influence MSM who are uninfected with HIV to participate in risky sexual behavior- a phenomenon called ‘AIDS treatment optimism’ [6]. HIV-infected MSM may also employ additional risk reduction strategies, such as choosing the receptive rather than the insertive role in anal intercourse, or performing oral rather than anal sex- a phenomenon called ‘strategic positioning’ [7].

Nevertheless, these strategies may not provide full protection. As HIV-incidence among MSM in Israel is increasing and HIV-infected MSM are the potential reservoir for further HIV-transmissions in the gay community, it is therefore necessary to study their knowledge, attitudes and practices in order to understand the dynamics of HIV transmission in Israel and to design targeted interventions for HIV-infected MSM.

## Methods

This cross-sectional study compared the knowledge, attitudes and sex-practices of HIV-infected MSM with that of HIV-uninfected MSM. The study instrument included anonymous questionnaires which were distributed in 2015 in AIDS treatment centers, and gay-related venues, events for HIV-infected individuals and on the internet (Additional file 1). Electronic banners advertising the survey were distributed in gay social network sites. MSM who clicked the banners were referred to web-questionnaires, where participants were asked different questions, depending on their HIV status, type of sex partners and sexual behavior. No financial incentive was offered.

The study size was calculated for a minimal difference of 2–3% in number of UAI between HIV-infected and non-HIV infected MSM who attend AIDS treatment centers. Study size for each arm was calculated with 5% type I error and 95% type II error, suggesting a minimum of 202 participants in each arm.

HIV-infected MSM were included if they had been treated with ART longer than one year and had visited one of the AIDS treatment centers in central Israel more than once, having been tested for CD4 count and plasma viral load, and if they had also reported that they had performed anal sex with another man during the last year. HIV-uninfected MSM were included if they had had anal sex with at least one other male partner during the last year, and had reported they were not known to be infected with HIV, as per a test performed within the last year. MSM younger than 18 years of age, and those who had not been living in Israel for more than one year were excluded.

Independent variables included demographic attributes, knowledge about HIV-transmission, attitudes towards condoms and harm reduction strategies, party drugs and condom-use as well as the reasons for not using condoms, sexual behavior in the last six months with different types of partners and additional risk behaviors. Dependent variables included self-reported HIV sero-status and UAI.

*Statistical analysis*: Characteristics of HIV-infected MSM were compared with those of HIV-uninfected MSM, and also between MSM who performed UAI in the last six months with those who did not. Univariate analysis was performed using the chi-square test for categorical variables and using the Student *t*-test for continuous variables in cases in which they were normally distributed or the Mann–Whitney test for variables which were not distributed normally. *P* values lower than 5% were regarded as statistically significant. Variables which were statistically significant in the univariate analysis were included in the multivariate analysis in the logistic regression to identify attributes predicting UAI after excluding collinearity, such as party drugs and alcohol.

## Results and discussion

This study included 300 HIV-infected and 1299 uninfected MSM who completed the questionnaires. Of the HIV-infected MSM, 185 (61.9%) were recruited electronically and 114 (38.1%) completed manual questionnaires, while among HIV-uninfected it was 1068 (82.2%) and 231 (17.8%), respectively. HIV-infected MSM were less educated than HIV-uninfected, more likely to self-identify themselves as gay men and more commonly reported previous sexually transmitted diseases (STD) diagnoses (Table 1). They were also more aware of the beneficial effect of ART in preventing HIV transmission. HIV-infected MSM were more likely to perform UAI than HIV-uninfected MSM, yet when they did, it was commonly performed with other HIV-concordant sexual partner/s. HIV-infected MSM were more likely to meet their sexual partners in saunas, clubs or through introduction by friends than HIV-uninfected participants. They used party drugs more commonly and were involved in additional risky sexual behaviors, such as earlier sexual debut and sex for money. In addition, they used more anti-depressant drugs in the last 6 months and reported dissatisfaction with their own health in comparison to HIV-uninfected MSM.

**Table 1:** Characteristics of men who have sex with men, by HIV status

Characteristic		HIV-infected *N* = 300 (%)	HIV-uninfected *N* = 1299 (%)	*P*
Demographic	Age in years (mean ± Sd)	35.0 ± 9.6	33.9 ± 9.6	0.05
	Israeli born	282 (86.2)	1157 (89.0)	0.06
	Less than average income^a^	181 (65.4)	790 (68.5)	0.1
	Jew	215 (93.5)	1232 (95.3)	0.2
	University education^a^	164 (55.2)	823 (63.4)	<0.001
	Self-identified gay	273 (96.1)	1446 (85.6)	<0.001
Previous STD diagnoses	Syphilis	72 (24.1)	29 (2.2)	<0.001
	Hepatitis C	10 (3.3)	6 (0.5)	0.002
	Hepatitis B	10 (3.3)	21 (1.6)	0.06
	Any STD^a,b^	171 (57.2)	401 (30.9)	<0.001
Knowledge about HIV transmission	Treated HIV-infected individual has low risk of transmission	198 (76.4)	444 (41.8)	<0.001
	In case the condom breaks, I will recommend PEP to my partner	175 (67.8)	737 (69.3)	0.7
	HIV infected man can perform UAI with serocon-cordant partner	125 (48.1)	332 (31.2)	<0.001
Reasons for not using condoms in the last 6 months	I was too horny	27 (52.9)	236 (54.3)	0.9
	I had a sero-concordant partner	35 (68.6)	169 (38.9)	<0.001
	I was under influence of substances	10 (21.7)	81 (18.6)	0.6
	Each partner is responsible for his own health	15 (31.3)	42 (9.7)	<0.001
	Condom breaks the intimacy	21 (45.7)	157 (36.1)	0.2
	I am sick of using condoms	19 (41.3)	151 (34.7)	0.4
	I am losing my erection	12 (26.1)	117 (26.9)	1.0
Raising HIV issue before/ during sex	HIV issue is always been raised before sex	245 (77.9)	1027 (75.6)	0.5
	I raise the issue of HIV	80 (40.6)	324 (39.6)	1.0
Where do you mostly meet your sex partners?	Phone application	210 (85.7)	942 (86.7)	0.6
	Facebook	25 (0.8)	140 (12.9)	0.4
	Park	21 (9.1)	52 (4.8)	0.02
	Sauna	33 (14.3)	74 (6.8)	<0.001
	Club	46 (19.5)	115 (10.6)	<0.001
	Friends	34 (14.5)	91 (8.4)	0.006
	Gym	13 (5.7)	27 (2.5)	0.02
Substances in the last 6 months	Use party drugs	197 (75.5)	622 (57.5)	<0.001
	Alcohol	118 (59.0)	546 (89.2)	<0.001
Additional risky sexual behavior	Age first sexual debut (years)	17.0 ± 4.4	18.4 ± 4.9	<0.001
	Ever been paid for sex	49 (18.8)	140 (13.5)	0.02
	I take more risks when I am abroad	32 (16.6)	85 (10.6)	0.03
Psychiatrics	Medical use of anti-depressant drugs in the last 6 months	80 (31.3)	142 (13.7)	<0.001
General subjective health status	Dissatisfied with my own health^c^	28 (11.1)	57 (5.5)	0.003
	I am healthier than my friends	64 (25.1)	384 (37.0)	<0.001

*STD* Sexually transmitted infections

*PEP* Post exposure prophylaxis

*UAI* Unprotected anal intercourse

^a^Adjusted for age

^b^Syphilis, Neisseria gonorrhea, Chlamydia trachomatis, genital warts, hepatitis B or C

^c^Dissatisfied vs. satisfied

Of all MSM who were in committed relationships, HIV-infected MSM were more likely to have sero-discordant/unknown HIV-status steady partner/s than HIV-uninfected MSM. HIV-infected were less likely to perform UAI with their sero-discordant/unknown HIV-status steady partner/s than HIV-uninfected MSM. In cases in which UAI was performed with their sero-discordant/unknown HIV-status steady partner/s, HIV-infected MSM tended to take the receptive role (Table 2).

**Table 2:** Unprotected anal intercourse (UAI) by HIV status and type of partner

Variable		HIV infected *N* = 300	HIV uninfected *N* = 1299	*P*
Steady partner	Number of men with steady partner N (% of all men)	104 (34.7)	448 (34.5)	1.0
	Sero-discordant/unknown-status steady partner N (% of all men with steady partner)	69 (66.3)	63 (14.1)	<0.001
	UAI with sero-discordant/unknown-status steady partner N (% of all sero-discordant/unknown-status steady partners)	26 (37.7)	33 (52.4)	<0.001
	Receptive UAI N (% of all UAI with sero-discordant/unknown-status steady partner)	24 (92.3)	23 (69.7)	0.04
Sex buddy	Number of men with sex buddy N (% of all men)	117 (39.0)	525 (40.4)	0.9
	Sero-discordant/unknown-status sex buddy N (% of all men with sex buddy)	29 (24.7)	253 (48.2)	<0.001
	UAI with sero-discordant/unknown-status sex buddy N (% of all sero-discordant/unknown-status partners)	8 (27.6)	98 (38.7)	0.01
	Receptive UAI N (% of all UAI with sero-discordant/unknown-status sex buddy)	7 (87.5)	47 (48.0)	0.01
Casual partner	Number of men with casual partner N (% of all men)	217 (72.3)	912 (70.2)	0.7
	Sero-discordant/unknown-status casual partner N (% of all men)	51 (23.5)	757 (80.3)	<0.001
	UAI with sero-discordant/unknown-status casual partner N (% of all sero-discordant/unknown-status casual partners)	12 (23.5)	182 (24.0)	0.8
	Receptive UAI N (% of all UAI with sero-discordant/unknown-status casual partner)	10 (83.3)	65 (35.7)	<0.001

Of all MSM who had sex buddy/ies, HIV-infected MSM were less likely to have sero-discordant/unknown HIV status partner/s than HIV-uninfected MSM. HIV-infected MSM reported less UAI with sero-discordant/unknown HIV status sex buddy/ies than HIV-uninfected MSM. In cases in which UAI was performed with their sero-discordant/unknown HIV-status sex buddy/ies, HIV-infected MSM were more likely to take the receptive role.

Of all MSM who had casual partner/s, HIV-infected MSM were less likely to have sero-discordant/unknown HIV-status casual sex partner/s than HIV-uninfected MSM. No statistical difference was found in the rate of UAI with sero-discordant/unknown HIV-status partner/s between HIV-infected and uninfected MSM. In cases in which UAI was performed with sero-discordant/unknown HIV-status casual sex encounter/s, HIV-infected MSM were more likely to take the receptive role.

Generally, HIV-infected MSM were less likely to perform UAI with sero-discordant/unknown HIV-status encounter/s than HIV-uninfected (39 [12.1%] vs. 232 [17.9%], respectively, *p* = 0.02). The rate of UAI among HIV-infected MSM with their sero-discordant/unknown HIV-status encounter/s was associated with the type of partnership. For example, UAI were more commonly performed with their sero-discordant/unknown HIV-status steady partner/s (66.3%), followed by sex-buddies (24.7%) and lowest with casual partner/s (23.1%), *p* = 0.02.

Of all HIV-infected, 109 (66.5%) reported their last viral load. Of those, 89 (81.7%) were undetectable and 20 (18.3%) had more than 50 copies. There were no differences in UAI between those who achieved viral suppression and those who did not (data not shown).

MSM in this study who performed UAI were more commonly younger and more likely to identify themselves as gay men than those who use condoms consistently and they also had more previous STD than those who used condoms constantly (Table 3). Their knowledge about post exposure prophylaxis (PEP) was inferior, yet they were more likely to raise the issue of HIV before sex, had more steady partners and more commonly used party drugs, alcohol and erectile dysfunction medications. In addition, they had earlier first sexual debut than those who used condoms. In the multivariate analysis, insufficient knowledge about PEP, raising the issue of HIV before/during sex, having a steady partner, reporting of a more common use of party drugs or erectile dysfunction medications and being at an early age for the first sexual debut was associated with UAI. HIV-infection was not found to predict UAI in the multivariate analysis.

**Table 3:** Univariate and multivariate analyses of sexual behaviors of men who have sex with men, by condom use

Characteristic		Univariate analysis			Multivariate analysis	
		UAI *N* = 532 (%)	Always condom *N* = 1066 (%)	*P*	OR (95% CI)	*P*
Demographic	Age in years (mean ± Sd)	34.0 ± 9.8	33.7 ± 10.4	0.04	1.0 (0.9–1.1)	0.9
	Israeli born	480 (90.2)	969 (91.2)	0.6		
	Less than average income^a^	303 (61.7)	668 (60.4)	0.5		
	Jew	487 (94.9)	960 (95.0)	0.9		
	University education^a^	313 (63.7)	674 (61.0)	0.1		
	Self-identified gay	472 (95.0)	850 (86.4)	<0.001	2.2 (0.9–5.5)	0.07
Previous STD diagnoses	HIV-infected	126 (23.7)	223 (20.1)	0.01	1.2 (0.8–1.3)	0.09
	Neisseria gonorrhea	126 (23.7)	138 (12.9)	<0.001		
	Syphilis	15 (9.6)	50 (4.7)	<0.001		
	Hepatitis B	17 (3.2)	14 (1.3)	0.02		
Knowledge about HIV transmission	Treated HIV-infected individual has low risk of transmission	240 (51.3)	402 (47.1)	0.1		
	In case the condom breaks, I will recommend PEP to the partner	287 (61.2)	625 (73.4)	<0.001	2.7 (2.0–3.6)	<0.001
Raising HIV issue before/ during sex	HIV issue is usually being raised before sex	266 (50.1)	415 (39.1)	<0.001	1.6 (1.4–2.6)	0.01
Type of partner	Steady	378 (71.1)	236 (22.1)	<0.001	15.3 (10.1–23.7)	<0.001
	Sex buddy	227 (42.7)	415 (38.9)	0.1		
	Casual	385 (72.4)	796 (74.7)	0.2		
Where do you meet your sex partners?	Phone applications	399 (85.3)	761 (88.2)	0.6		
	Facebook	61 (13.0)	104 (12.3)	0.7		
	Park	24 (5.1)	49 (5.8)	0.6		
	Sauna	39 (8.3)	68 (8.0)	0.9		
	Club	54 (11.4)	107 (12.6)	0.6		
	Friends	45 (9.6)	80 (9.4)	0.9		
	Gym	10 (2.1)	30 (3.6)	0.2		
Substances/ drugs in the last 6 months	Party drugs	316 (65.7)	503 (58.4)	0.005	2.0 (1.2–3.2)	0.006
	Alcohol	281 (87.8)	383 (77.8)	<0.001		
	erectile dysfunction medications	116 (35.8)	107 (21.8)	<0.001	2.0 (1.2–3.5)	0.007
Additional risky sexual behavior	Age first sexual debut (years)	17.5 ± 4.6	18.5 ± 4.9	0.001	1.2 (1.1–1.2)	0.01
	Was paid for sex	85 (18.7)	109 (12.8)	0.05		
	I take more risks abroad	134 (26.9)	258 (23.3)	0.08		
Psychiatrics	Medical use of anti-depressant drugs in the last 6 months	72 (15.2)	150 (18.3)	0.2		
General subjective health status	Dissatisfied with my own health^b^	26 (5.5)	59 (7.2)	0.2		

*OR* Odds ratio

*CI* Confidence interval

*STD* Sexually transmitted infections

*PEP* Post exposure prophylaxis

*UAI* Unprotected anal intercourse

^a^Adjusted for age

^b^Dissatisfied vs. satisfied

In this study, 12.1% of the HIV-infected and 17.9% of the HIV-uninfected MSM reported UAI with a sero-discordant/unknown HIV-status partner during the previous six months, lower than the range of 13–51% UAI during the last year, as reported in a literature review [8]. HIV-infected MSM frequently employed the ‘sero-sorting’ and ‘sero-positioning’ strategies, while using condoms more commonly than HIV-uninfected MSM during anal intercourse with their sero-discordant/unknown HIV-status sex partners and selecting the receptive role when preforming UAI. HIV-infected MSM also demonstrated a better knowledge regarding HIV prevention and transmission than MSM who were not infected.

Although HIV-infected MSM in this study were involved in behaviors which were associated with a greater sexual risk, such as the use of party drugs, erectile dysfunction medications and early first sexual debut, they were generally more compliant in using condoms with their HIV sero-discordant/unknown HIV-status partners, as also found in another study [9]. However, they reported that they expected that the responsibility of using a condom would be shared by their partner/s. In cases in which the issue of HIV was not raised before sex, then HIV-infected MSM might have assumed that their partner was either careless or sero-concordant, and chose to perform UAI. Shifting the responsibility to the sex partner was found to be associated with additional risky sexual behaviors [10] and also with ‘sex on premises venues’ or anonymous sex, when there are fewer bonds of social obligations, implying reciprocal care of the sexual encounter/s [11]. The results of this study demonstrate the complexity of disclosure of their HIV status to their partners. On one hand, HIV-infected MSM are probably aware that they are morally and possibly legally obliged [12] to inform their partner of their sero-status, or at least use a condom in anal sex, especially if they are not treated or have not achieved undetectable viral load. On the other hand, they are concerned that the HIV-uninfected partner/s may reject them upon disclosure or that their confidentiality is breached [13]. HIV prevention should therefore include social support for HIV-infected MSM to encourage them to disclose their sero-status to their partner, or use a condom with HIV-discordant/unknown HIV-status partner/s to make personal responsibility more salient, or to adhere to their ART. Concomitantly, HIV-uninfected MSM should also be encouraged to raise the issue of HIV sero-status with their sex partner/s, and be informed that sex with HIV-infected partner/s is safe as long as proper biological or mechanical protection is used, rather than avoiding sex partners whose HIV sero-status is positive [14, 15].

A minority of HIV-infected MSM performed UAI with a sero-discordant partner although their viral load was detectable. Yet, in most cases HIV-infected MSM performed UAI with sero-concordant sexual partner/s. In these cases they may be exposed to coinfection with other STD, which may complicate and accelerate HIV disease [16]. It has recently been demonstrated that the rise of syphilis in Israel was fueled by UAI between HIV sero-concordant sex partners [17].

In cases in which HIV-infected MSM in this study performed UAI with HIV sero-discordant/unknown partner/s, they commonly used strategic positioning, while choosing the receptive role in anal sex. These calculated risk-taking strategies may reduce, yet not eliminate, HIV transmission. However, these efforts are not always fully appreciated by those who provide medical and social services for HIV-infected MSM. Providers should rather understand the difficulty of HIV-disclosure, the pursuit of HIV-infected MSM to reach intimacy with the sex partner, and their fear of being stigmatized or rejected, and also be aware of the psychological context that frames condom-use [18]. Providers should maintain an open environment in their clinics when treating HIV-infected MSM, in which patients can appraise their desires and their actions, and evaluate their sexual behaviors and reasons why they engaged in risk-behaviors [19].

Most of the participants in this study were not aware of the HIV sero-status of their casual partners. In the majority of those cases, mutual HIV status was not raised before or during sex, not allowing the encounters to negotiate safer sex practices or perform strategic positioning. Interestingly, HIV-infected MSM had performed more UAI with their sero-discordant/unknown HIV-status steady partners, and less with their sero-discordant/unknown HIV-status sex buddies or casual partners. This is an additional finding demonstrating the difficulty in maintaining a constant condom use for longer periods with a regular sex partner.

HIV-infected MSM were more likely to use psychiatric drugs and reported that their general health status was inferior to that reported by HIV-uninfected MSM. It is not known whether they used psychiatric medications because they were infected, or that they felt more comfortable using these medications than HIV-uninfected participants did, or that maybe they had used the drugs before they were infected [20]. AIDS treatment centers should employ multidisciplinary approach to HIV-infected MSM, which includes mental care and effective response to additional medical needs of HIV-infected MSM.

The results of this study should be weighed against recent publications describing the success of ART in HIV-infected MSM and its role in preventing HIV transmissions. Although UAI has traditionally been used as a key component in defining risky sexual behavior, there is a growing, largely coherent, body of evidence that HIV-infected MSM who adhere to ART and are virally suppressed may render HIV transmission negligible [21, 22]. This approach can be reinforced by using additional risk-reduction strategies, such as sero-adaptive sex behaviors, strategic positioning, or withdrawal [23]. Notwithstanding, there is also an emerging consensus as to the effectiveness of pre-exposure prophylaxis (PrEP), a course of ART that can be used by HIV-uninfected men to prevent seroconversion [24]. PrEP has become more popular among MSM, including those is Israel, and it is the authors’ impression that HIV-uninfected MSM are making efforts to get the treatment (purchasing through the internet, during a visit to developing countries, or falsely claim that they were exposed to HIV and ask for PEP, while actually using it as PrEP). The success of these biomedical innovations may justify a fresh perception of UAI by researchers and providers, whether UAI accurately defines risk behavior.

This is the first study in Israel describing knowledge, attitudes and behaviors of HIV-infected MSM, yet it is subject to several methodological limitations. First, the cross-sectional design of the study limits the establishment of causality. A few examples: are HIV-infected MSM involved in risky sexual behavior and therefore become infected or that they were they involved in risky sexual behavior after becoming infected; Did HIV-infected MSM change their role in anal sex to the receptive position because of their HIV-infection in order to decrease the risk of HIV-transmission or that they practiced receptive anal sex prior to their HIV-infection? Second, we used a convenience sample of HIV-infected, which is subject to selection bias and it limits generalizability. Yet, this method is accepted in studies in hard-to-reach populations focusing in sensitive issues, such as sexual behavior, especially among HIV-infected MSM, as they are stigmatized. In order to encourage recruitment and increase heterogeneity, MSM (both HIV-infected and uninfected) were approached both in medical and non-medical settings, and the questionnaires were also available electronically in a popular gay-related internet site. Third, all data in this study were self-reported and as such potentially subject to social desirability or recall bias. In order to minimize this bias, the information was collected anonymously and pertained only to events which occurred during the last year.

## Conclusions

In summary, HIV-infected MSM performed UAI less frequently with their sero-discordant/unknown HIV-status encounters than HIV-uninfected MSM. HIV-infected MSM tended to used sero-adaptive strategies to reduce the potential risk of HIV-transmission to their sero-discordant/unknown HIV-status partner/s. In those cases in which HIV-infected MSM performed UAI, they reported that a shared responsibility by both partners was expected. In order to increase mutual sero-disclosure, HIV-infected MSM should be encouraged to discuss their HIV status. AIDS treatment center should include mental support for HIV-infected MSM and instruct the patients how to practice safe sex and encourage the discloser of their HIV status to their partners.

## Additional file

Additional file 1: Study questionnaire. (DOCX 35 kb)

## Abbreviations

ART: Anti-retroviral treatment
HIVI: HIV infected
HIVU: HIV uninfected
MSM: Men who have sex with men
PEP: Post exposure prophylaxis
PrEP: Pre-exposure prophylaxis
STD: Sexually transmitted diseases
UAI: Unprotected anal intercourse
