# Supplementary material for: HIV infected men who have sex with men in Israel: knowledge, attitudes and sexual behavior
Source: BMC Infect Dis. 2017 Oct 12;17:679. doi: 10.1186/s12879-017-2782-1 (PMC5639789; doi:10.1186/s12879-017-2782-1)
Supplement: Additional file 1: — Study questionnaire. (DOCX 35 kb) [file 12879_2017_2782_MOESM1_ESM.docx]

**Additional file 1**

**Study questionnaire**

Demography:

Year of birth

Country of birth, year of arrival in Israel

Occupation (soldier, student, unemployed, employed)

Monthly income

HIV status

How were you infected (sexual contact with men, sexual contact with women, intr-venous drug injection, blood transfusion, mother to child, do not know)

Year of infection (estimated)

Year of diagnosis

Where were you detected as HIV positive (by my personal doctor, hospital, AIDS task force, military, other)

How many people know about your HIV status? (no one knows, close friends, parents, family, colleagues from work/university, everybody knows)

Sexual orientation

Whom you make sex with? (men only, both men and women, women only)

Who knows you make sex with men? no one knows, close friends, parents, family, colleagues from work/university, everybody knows)

Health status

What are your last CD4 and viral load count?

When was your last CD4/viral load count?

Have you been diagnosed with any of those pathogens (tuberculosis, hepatitis B, hepatitis C, Candida, condyloma)

Have you been diagnosed with malignancies (after you were diagnosed with HIV)? (lymphoma, Kaposi sarcoma, other: ___)

Sexual relations (sex=oral, vaginal, anal sex):

How old were you in the first time you had sex with men?

How old were you when you had your first anal sex with men?

If you had sex with women- how old were you in the first time you had sex with women?

Do you have a steady partner currently?

If so, how long have you been together?

What is the HIV status of your current steady partner? (Positive, negative, do not know)

Have you had anal sex without a condom with your steady partner in the last 6 months? (Often, sometimes, rarely, never)

When you had anal sex without condom with your steady partner, were you – top? bottom? Both (versatile)

Have you had casual sex partners in the last 6 months?

If yes, with how many?

Have you had oral sex with ejaculation them? (they performed oral sex on you and you came in their mouth)? Often, sometimes, rarely, never

Have you had anal sex without a condom with your casual partners? (Often, sometimes, rarely, never)

With how many casual partners have you had anal sex without a condom in the last 6 months?

Have you had anal sex with a casual partner in the last 6 months without knowing his HIV status and came inside?

If you had anal sex without a condom with both casual and steady partner/s, is there any difference in the sexual habits? (with my steady partner I am more top than with casual, with my steady partner I am more bottom than with the casual, it does not make any difference)

Is the HIV status discussed before or during sex in the first sexual contact? (Often, sometimes, rarely, never).

If so, who raises the issue? (usually me, usually my partner/s, sometimes me and sometimes my partner/s)

Do you normally inform your partner/s that you are HIV positive in the first sexual contact? (always, sometimes, rarely, never)

What is the reason that you did not disclose your partner that you are HIV-positive?

| Not true | Sometimes true | Usually true | True |  |
| --- | --- | --- | --- | --- |
|  |  |  |  | I feel ashamed that I am HIV-positive |
|  |  |  |  | I was afraid he would refrain to have sex with me |
|  |  |  |  | I wanted to tell him, but I did not know how |
|  |  |  |  | I was afraid he would transmit the information to other/s |

What was the reason you did not use a condom in the last 6 months? (You can check more than one)

| Not true | Sometimes true | Usually true | True |  |
| --- | --- | --- | --- | --- |
|  |  |  |  | I was too horney and did not care |
|  |  |  |  | My sexual partner identified he was HIV-positive |
|  |  |  |  | I assumed that my sexual partner was HIV-infected by his behavior |
|  |  |  |  | I was "stoned" or drunk |
|  |  |  |  | Because I was infected, I did not care to transmit the infection to other/s |
|  |  |  |  | I needed the intimacy and the direct contact |
|  |  |  |  | I am sick and tired of using condom all the time |

Where do you meet most of your sexual partners?

| never | rarely | sometimes | Often |  |
| --- | --- | --- | --- | --- |
|  |  |  |  | Internet |
|  |  |  |  | Virtual social network |
|  |  |  |  | Parks, beach |
|  |  |  |  | Bath house, dark room |
|  |  |  |  | Bars, clubs |
|  |  |  |  | Friends |
|  |  |  |  | Gym |
|  |  |  |  | Social event of the AIDS task force |

Do you use substances before/while sex?

| never | rarely | sometimes | Often |  |
| --- | --- | --- | --- | --- |
|  |  |  |  | Alcohol |
|  |  |  |  | Grass, Hash |
|  |  |  |  | PCP, GHB, Special K |
|  |  |  |  | Ecstasy, tina, crack, cocaine, gathinon, speed, Ritalin |
|  |  |  |  | Poppers, glue |
|  |  |  |  | Viagra, cialis, Levitra |
|  |  |  |  | IV drug use |

Have you been in a group sex in the last 6 months? (yes, no)

What is the preferred age of your sexual contacts (younger than me, about my age older than me)

Have you ever paid for sex? (if yes- often, sometimes rarely, once)

Have you ever received money (or other valuable) for sex? (if yes- often, sometimes rarely, once)

Have you changed your sexual habits one you were diagnosed with HIV?

| Not true | Sometimes true | Usually true | True |  |
| --- | --- | --- | --- | --- |
|  |  |  |  | I try to reduce the time I am bottoming |
|  |  |  |  | I try to suck more than being sucked |
|  |  |  |  | When I am topping, I try more to use condom |
|  |  |  |  | When I am bottoming, I try more that my partner use a condom |
|  |  |  |  | I did not use my sexual habits |
|  |  |  |  | I do reduce the frequency I make sex |
|  |  |  |  | I am less careful than I used to be before diagnosis |
|  |  |  |  | I must use drug to function properly in bed |

When you travel abroad, do you change your cautiousness regarding condom use? (yes- I am less careful, no- I am more careful, I did not change my sexual habits)

Attitudes

| Not true | Sometimes true | Usually true | True |  |
| --- | --- | --- | --- | --- |
|  |  |  |  | Do you believe that HIV positive man can have receptive anal sex without transmitting the virus? |
|  |  |  |  | Do you believe that HIV positive man can have insertive anal sex without transmitting the virus? |
|  |  |  |  | Is condom efficient in preventive STD/HIV? |
|  |  |  |  | If the condom breaks while you have anal sex, will you recommend your partner to ask his doctor for post-exposure prophylaxis? |
|  |  |  |  | Do you believe that HIV-positive men can have unprotected anal sex with other HIV-positive men? |
|  |  |  |  | Because there is efficient HIV treatment, I can have sex without condom |
|  |  |  |  | If my partner does not insist of having a condom, we shall continue without it |
|  |  |  |  | I tend to think more often of death once I was diagnosed with HIV |
|  |  |  |  | I am more afraid of dying since I was diagnosed with HIV |

ART

Are you currently treated with ART?

If yes, for how long?

What is your current medical treatment?

What is your normal adherence (very good, good, fair, bad)

Do you come to the AIDS centers every 3 months? (Yes, mostly, no)

Why you do not comply with your medical treatment (side effects of the drugs, I do not remember to take the drugs, I do not take the drug to forget I am HIV-positive, it is OK to forget sometimes, I do not have the drugs, I cannot afford the drugs)

Have you changed the treatment regimen (yes, because I had side effects; yes, because my viral load increased or just did not reduced as expected; yes, because the new regimen was more convenient; yes, other cause)

Do you have drug resistant virus? (I do not know, no, partial resistance, full resistance)

Are you treated with prophylactic therapy other than ART? (If yes, which? For how long?)

Treatment in the clinic (other than AIDS clinics) and hospitals

Does your family doctor know you are HIV-positive?

Do you tell your other doctors that you are HIV-positive? (Yes, all of my other doctors; yes, if I find it relevant; no)

What is the reason/s you do not tell the other doctors you are HIV-positive? (I am afraid my privacy will not be respected, I am afraid my the quality of the treatment will be affected)

Can you describe a medical situation where you felt uncomfortable as a HIV-positive patient? Describe: ____

Which of the following professions is the most sensitive for HIV-positive? Dentist, dermatologist, surgeon, family physician, endocrinologist, gastroenterologist, orthopedic, ophthalmologist, ENT, other___)

Do you think you are getting worse medical treatment than HIV-negative patients? (yes, no)

Have you been treated in the last year with treatments which are not covered by your health insurance scheme related to your HIV condition? (yes, no)

What is the estimated annual expenditure of HIV-related treatment?

Have you consulted a psychiatrist or been prescribed antidepressant medications in the last 6 months? (yes, no)

Are you satisfied with the services you get from your insurer (very satisfied, mostly satisfied, somehow satisfied, not satisfied)

Health determinants

Grade your health status (excellent, good, fine, not good, bad)

Do you have any mood disorders? (depression, nervousness, cries a lot, anxiety, obsessive thoughts, sleeping disorders, sadness)

Are you sick more often than other HIV-positive men (yes, no)

Do you currently perform physical activity (more than 30 minutes more than 3 times a week)? (yes, no)

Treatment experience in the AIDS center

Are you satisfied with the services you get from your AIDS center? (very satisfied, mostly satisfied, somehow satisfied, not satisfied)

Are you satisfied from the way the AIDS center keeps your privacy? (very satisfied, mostly satisfied, somehow satisfied, not satisfied)

Have you changed your health clinic since you started treatment? (yes, no)

If you did, what was the reason? (changing address, being unsatisfied with the previous treatment center, because I was not satisfied with one of the physician there, recommendations of other HIV-positive friends, other: ___)

Did any of the physicians in the I|DS clinic have provided you training regarding safe sex? (yes, no)

Was it beneficial (yes, no)

Has any of the physicians has initiated in the last year the following? Referred you to have STD tests; run a conversation about using psychoactive substances; conversation about sexual function; conversation about mood)

Do you feel comfortable to consult your doctor at the AIDS regarding these issues? (yes, no)

Do you feel the AIDS treatment center provides a responsive and suitable response to HIV-positive patients? (an open question)
